# Compatibility of Brazilian Strains of *Trichoderma afroharzianum* with Various Agricultural Inputs Under In Vitro Conditions

**DOI:** 10.3390/jof11110812

**Published:** 2025-11-16

**Authors:** Eder Marques, Moisés Rodrigues Silva, Wanessa Mendanha Soares, Keren Hapuque Mendes de Castro, Joyce Gonçalves da Silva, Karolyne Campos da Silva, Marcos Gomes da Cunha

**Affiliations:** 1 Plant Pathology Research Center, Phytosanitary Sector, College of Agronomy, Federal University of Goiás, Samambaia Campus, Av. Esperança s/n, Goiânia 74690-900, Goiás, Brazilmgc@ufg.br (M.G.d.C.); 2 Plant Breeding Sector and Plant Pathology Research Center, College of Agronomy, Federal University of Goiás, Samambaia Campus, Av. Esperança s/n, Goiânia 74690-900, Goiás, Brazil; moises.rodrigues@discente.ufg.br

**Keywords:** antagonistic effect, conidia germination, microbial products, pesticides, spray mixture, sustainable agriculture

## Abstract

*Trichoderma afroharzianum* is increasingly used in commercial bioformulations for plant disease management in Brazil, yet information about its compatibility with agricultural inputs remains scarce. This study evaluated the in vitro interactions between four Brazilian strains of *T. afroharzianum* and 32 inputs from different classes, including fungicides, herbicides, insecticides, and microbial products. Mycelial growth and conidial germination were analyzed to identify potential incompatibilities that could compromise the fungus’s performance in combined applications. In the first bioassay, 16 products inhibited mycelial growth by more than 67%. In the second, which simulated spray solution contact, most products (12 out of 16) did not significantly affect conidial germination (>76%). However, the bionematicide based on *Bacillus amyloliquefaciens* and the fungicide Picoxystrobin + Benzovindiflupyr reduced germination to below 9% and 14%, respectively. Fungicides containing Picoxystrobin + Prothioconazole and Trifloxystrobin + Tebuconazole showed strain-dependent inhibition. These results provide the first detailed assessment of the compatibility of Brazilian *T. afroharzianum* strains with a broad range of agricultural inputs and can support safer use of biocontrol agents in integrated and sustainable crop protection strategies.

## 1. Introduction

The concept of sustainable food systems (SFS) has gained increasing attention as a framework to balance food production, environmental preservation, and human health [1,2]. Despite this, the global use of agrochemicals continues to expand, posing significant challenges to environmental integrity through contamination of air, soil, and water, and to biodiversity and human well-being [3]. Fungal plant diseases are among the main causes of yield losses worldwide, with global damage exceeding US$ 220 billion annually [4]. These pressures underscore the need for effective and safer alternatives to conventional fungicides and other chemical pesticides.

Biological control has emerged as a cornerstone of sustainable crop protection, particularly within integrated pest management (IPM) programs [5]. Among fungal biocontrol agents, the genus *Trichoderma* stands out for its broad range of antagonistic mechanisms, plant growth promotion, and capacity to adapt to diverse agroecosystems [6,7]. The first commercial formulation based on *T. harzianum* was registered in 1989 [8], and since then, several species have been developed for global use, including *T. asperellum*, *T. atroviride*, and *T. viride*. The species *T. afroharzianum* was described or reclassified in 2015 [9] and has a worldwide distribution. It is already included in commercial products. In Brazil, the market for biological inputs has expanded rapidly under the National Bio-inputs Program [10,11], with 112 *Trichoderma*-based products registered as of October 2025, including two of *T. afroharzianum* [12].

In agricultural practice, tank mixtures of agrochemicals are frequently adopted to improve operational efficiency, reduce application costs, and broaden the pest control spectrum [13,14]. However, combining biological and chemical products can lead to negative interactions that compromise microbial survival or activity, undermining the effectiveness of biocontrol agents [15]. Compatibility tests are therefore essential to identify which products can be safely combined without reducing the efficacy of beneficial microorganisms.

Previous studies have investigated the compatibility of *Trichoderma* species with agrochemicals, including *T. harzianum* [16,17], *T. viride* [18], and *T. asperellum* [19]. Nevertheless, information on *T. afroharzianum* remains limited. The only available study, conducted by Ramanagouda and Naik [20], evaluated a small number of products, without considering strain-level variation. Moreover, despite the inclusion of *T. afroharzianum* in Brazilian commercial bioformulations, to date, no studies have characterized the compatibility of local strains with agricultural inputs.

Considering the growing use of *T. afroharzianum* in bioproducts and the potential for contact with agricultural inputs in agricultural operations, understanding these interactions is critical to ensure successful application in IPM programs. Therefore, the objective of this study was to evaluate, under in vitro conditions, the compatibility between four Brazilian strains of *T. afroharzianum* and a wide range of agricultural inputs, including fungicides, herbicides, insecticides, and microbial products. This investigation provides new insights into the potential interactions that may affect the performance of *T. afroharzianum* in combined or sequential applications, contributing to safer and more sustainable crop protection strategies.

## 2. Materials and Methods

### 2.1. Origin of Trichoderma afroharzianum Strains

The four *T. afroharzianum* strains used in this study were previously obtained, molecularly identified, and characterized for their antagonistic properties in earlier studies, where they demonstrated high efficacy in in vitro antagonism against various plant pathogens, as well as the ability to suppress plant diseases [21,22,23,24]. The strains were selected based on their distinct ecological origins, in order to assess the response of isolates from different plant hosts to the agricultural inputs tested. Strains NPF 10 and NPF 11 were originally isolated as endophytes from sugarcane (*Saccharum officinarum*), whereas strains NPF 29 and NPF 30 were obtained from the rhizosphere of lemongrass (*Cymbopogon citratus*). This approach allowed us to compare the behavior of isolates with different ecological backgrounds regarding their compatibility with agricultural chemicals. They are maintained in the working collection of the Plant Pathology Research Center (NPF) under the following preservation conditions: Castellani method, 10% glycerol at 4 °C, and storage at −80 °C. For the bioassays, the strains were reactivated from storage by culturing on commercial potato dextrose agar (PDA) and incubated at 28 °C under a 12 h photoperiod for seven days.

### 2.2. Agricultural Inputs Evaluated in Bioassays

A total of 32 agricultural inputs from different classes were evaluated, including ten of microbiological origin, eleven insecticides, six fungicides, and five herbicides. In addition to representing distinct chemical classes, the selected agricultural inputs were chosen to encompass a variety of mechanisms of action against their target organisms. All products were analyzed within their respective expiration dates. Detailed information on the active ingredients, product classes, and concentrations used in the spray solutions for each treatment is provided in Table 1.

### 2.3. Effect of Agricultural Inputs on Mycelial Growth

In the bioassay aimed at evaluating the effect of agricultural inputs on the mycelial growth of *T. afroharzianum*, the incorporation method in molten PDA culture medium was used, as described by Celar and Kos [19]. The products were added to the commercial PDA medium while still liquid, at a temperature of 48 °C, in order to prevent thermal inactivation, at the respective doses established for each treatment, and poured into 55 mm diameter plastic Petri dishes. At the same time, suspensions of the four *T. afroharzianum* strains were prepared by washing seven-day-old cultures with sterile distilled water, followed by dilution of the concentrated suspension as needed. Conidia were quantified in a Neubauer chamber under optical microscopy, and the suspension was adjusted to a final concentration of 1 × 10^7^ conidia/mL.

A 0.5 µL aliquot of this suspension was inoculated at the center of each plate containing the products previously incorporated into the culture medium. The plates were incubated at 28 °C under a 12 h photoperiod until the controls (plates containing only the antagonist fungus) had completely colonized the surface of the medium, at which point the diameter of mycelial growth (cm) was measured using a millimeter ruler. The doses of chemical products (fungicides, herbicides, and insecticides), expressed as concentrations, are presented in Table 1. These doses were determined based on the information provided on the respective product labels, in accordance with the recommended application rates for field use. In the case of biological products, all were adjusted to a final concentration of 1 × 10^7^ conidia or CFU/mL. Each treatment was performed in triplicate, and the assay was conducted twice.

### 2.4. Simulation of Spray Solution and Effect of Inputs on Conidial Germination

In the assay aimed at evaluating the effect of agricultural inputs on the germination of *T. afroharzianum* conidia, 16 products were selected based on their high inhibition rates of mycelial growth (>67.3%) observed in the previous experiment. To simulate the spray solution, each product was diluted in sterile distilled water at the same concentrations described in Table 1. Suspensions of the four *T. afroharzianum* strains were then added to these solutions and adjusted to a final concentration of 1 × 10^6^ conidia mL^−1^. The mixtures containing both the products (25 mL) and fungal strains were kept under constant agitation at 150 rpm for 2 h. Subsequently, 10 µL aliquots of each suspension were pipetted onto 85 mm diameter plastic Petri dishes containing commercial PDA medium and evenly spread using a Drigalski loop. The control treatment consisted of plates with PDA medium inoculated with the same fungal strains but without the addition of agricultural inputs.

The plates were incubated at 28 °C under a 12 h photoperiod. Approximately 16 h after inoculation, conidial germination was evaluated under a light microscope at 40× magnification, considering germinated conidia as those exhibiting a germ tube length equal to or greater than the conidial diameter. For each treatment, 100 conidia were counted across random microscopic fields. The bioassay was performed with three replicates per treatment and repeated twice to ensure reproducibility.

The germination rate (%) was calculated using the following formula:

*CGI* = (*Gt*/*Gc*) × 100

where *Gt* is the germination rate observed in the treatment and *Gc* is the germination rate of the control.

### 2.5. Experimental Design and Data Analysis

The experiments were conducted in a completely randomized design with three replicates per treatment, following a 32 × 4 factorial scheme (32 agricultural inputs and 4 strains for the mycelial growth inhibition assay) and a 16 × 4 scheme (16 agricultural inputs and 4 strains for the conidial germination assay). Prior to statistical analysis, data were tested for normality (Shapiro–Wilk test) and homogeneity of variances (Levene test), and both assumptions were not met (*p* < 0.05). Therefore, a non-parametric clustering approach was adopted using the silhouette validation method implemented in R software version 4.5.0 [25]

Introduced by Rousseeuw [26], this method calculates the silhouette width for each observation, the mean silhouette width for each group, and the overall average silhouette of the dataset. The silhouette width compares intra-group cohesion (similarity of an observation to its own group) with inter-group separation (similarity to the nearest neighboring group). Values range from −1 to 1, with scores close to 1 indicating well-defined groups, values near 0 suggesting overlap between groups, and negative values indicating possible misclassification. This approach is particularly suitable for datasets that deviate from normality, providing a robust and distribution-free measure of group validity.

The construction of the silhouette *S*(*i*) is given by the following equation:

*S*(*i*) = ((*b*(*i*) − *a*(*i*)))/max{*a*(*i*),*b*(*i*)},

in which:

*a*(*i*) represents the average dissimilarity between object *i* and the other objects in the same group;

*b*(*i*) represents the smallest average dissimilarity between object *i* and the objects in other groups (i.e., the closest group).

The value of *S*(*i*) ranges from −1 to 1. Values close to 1 indicate that the observation has been correctly grouped; values close to 0 suggest that the observation lies near the boundary between two groups; and negative values indicate that the observation may have been incorrectly assigned to a group. The average *S*(*i*) across all observations is used to validate the groupings and determine the optimal number of clusters.

### 2.6. Calculation of the Mycelial Growth Inhibition Index and Heatmap Construction

The mycelial growth inhibition index (MGI) was calculated based on the data obtained in both experiments, using the following formula [19]:

MGI = [(C − T)/C] × 100,

in which:

C represents the diameter of mycelial growth in the control treatment (without application of agricultural inputs);

T represents the diameter of mycelial growth in the treatment with the application of agricultural inputs.

To visualize the overall effect of agricultural inputs on the mycelial growth of the four *T. afroharzianum* strains, a heatmap was generated using the *pheatmap* package in R software version 4.5.0 [25]. The data were first organized into a matrix containing the average mycelial growth inhibition (MGI) rates for each product–strain combination. Hierarchical clustering based on Euclidean distance was then applied to group inputs and strains according to similarity in inhibition profiles. The clustering results were interpreted using the silhouette values obtained in the previous analysis, where values close to 1 indicated well-defined groups, values near 0 suggested partial overlap between clusters, and negative values indicated potential misclassification. This combined approach allowed a clearer visualization of compatibility patterns, highlighting which agricultural inputs consistently promoted or inhibited fungal growth across strains.

## 3. Results

### 3.1. Analysis of Mycelial Growth in Bioassay with Products of Microbial Origin

Regarding the effect of the microbiological products evaluated on mycelial growth, a consistent pattern was observed among the biological replicates of *T. afroharzianum*, as analyzed using the silhouette validation method (Table 2). The average silhouette values of the evaluations were greater than 0.77. Treatments that promoted greater mycelial development—i.e., showed lower inhibition—included those based on *B. elkanii*, *B. japonicum*, *B. pumilus*, and *T. harzianum*. These products, along with the control, were included in group 3 (growth greater than 5.12 cm), with average silhouette values above 0.77, indicating good separation between groups. Group 2, which exhibited intermediate growth, included *P. aryabhattai* (in strains 1 and 2, with growths of 3.77 and 3.85 cm, respectively) and *B. methylotrophicus* (growth between 2.33 and 4.17 cm). In specific cases, for strains 3 and 4, the treatment with *P. aryabhattai* clustered with the control, recording growths of 5.12 and 5.02 cm, with average silhouette values of 0.86 and 0.81, respectively. The remaining products—*B. amyloliquefaciens*, *B. subtilis*, *B. subtilis* + *B. licheniformis*, and *B. velezensis*—formed group 1, which was characterized by the greatest inhibition of mycelial growth. The observed growth values were 0.00 cm; between 0.00 and 0.17 cm; from 0.3 to 0.67 cm; and between 0 and 1.48 cm, respectively.

### 3.2. Heatmap Analysis of Mycelial Growth Inhibition

**Figure 1:**
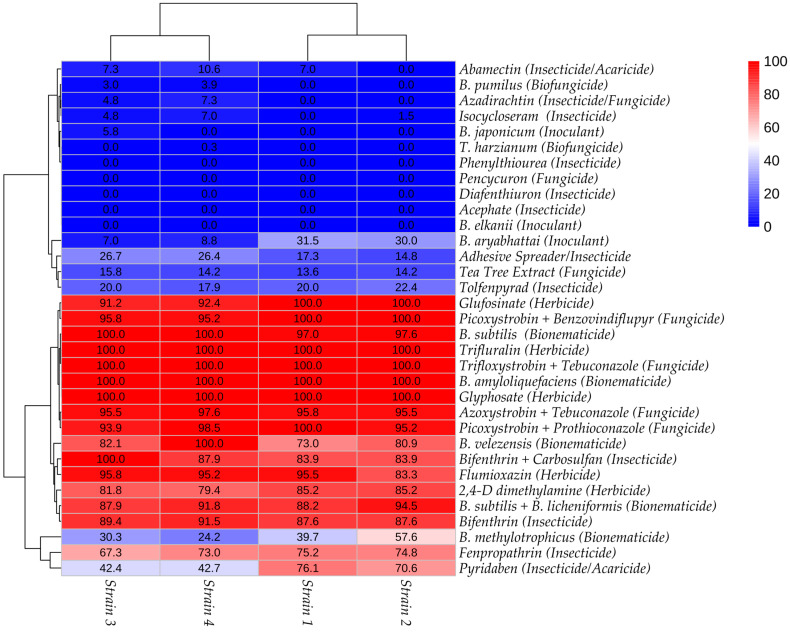
Heatmap constructed based on the average mycelial growth inhibition index (MGI) observed in the in vitro compatibility assay involving 32 agricultural inputs and four strains of *Trichoderma afroharzianum.*

The pattern of mycelial growth inhibition by biological treatments is clearly illustrated in the heatmap constructed using the mycelial growth inhibition index (MGI), as shown in Figure 1. In this representation, varying shades of blue (0 to 42.7%) and red (57.6 to 100%) indicate different levels of inhibition, allowing for a rapid visual assessment of the relative effectiveness of each treatment across the four strains of *T. afroharzianum*.

### 3.3. Analysis of Mycelial Growth in the Bioassay with Chemical Fungicides

In the bioassay analysis involving six chemical fungicides, a consistent pattern of mycelial growth inhibition was observed across the four *T. afroharzianum* strains, with an average silhouette score of 0.84, indicating good cluster formation (Table 3). Group 1, characterized by the lowest mycelial growth, included treatments with fungicides containing Azoxystrobin + Tebuconazole, Picoxystrobin + Benzovindiflupyr, Picoxystrobin + Prothioconazole, and Trifloxystrobin + Tebuconazole, with growth values ranging from 0.13 to 0.25 cm, 0.00 to 0.27 cm, 0.00 to 0.33 cm, and 0.00 cm, respectively. Group 2 was exclusively composed of the botanical fungicide based on tea tree extract, which resulted in only a slight reduction in mycelial growth, with values ranging from 4.63 to 4.75 cm. In contrast, the fungicide formulated with Pencicurom showed no inhibitory effect on any of the strains and was grouped with the control in Cluster 3. An overview of the results, along with the average mycelial growth inhibition indexes (MGI), is presented in the heatmap shown in Figure 1.

### 3.4. Analysis of Mycelial Growth in Bioassay with Chemical Insecticides

Most of the chemical fungicides evaluated exhibited similar effects across the four *T. afroharzianum* strains, with cluster analyses showing adequate support (mean silhouette values > 0.65; Table 4). Group 3, which showed the greatest mycelial growth (i.e., least inhibition), was consistent among biological replicates and included treatments with Abamectin + Cyantraniliprole, Acephate, Azadirachtin, Diafenthiuron, Isocycloseram, and the control, with values ranging from 4.92 to 5.5 cm. Tolfenpyrad was also classified in this group for strain 4 (4.52 cm), as well as Diafenthiuron for strains 3 and 4 (5.5 cm). Group 2 included treatments with intermediate inhibition levels, such as the adhesive spreader (4.03–4.68 cm), Tolfenpyrad in strains 1–3 (4.4, 4.25, and 4.4 cm), Pyridaben in strains 3 and 4 (3.17 and 3.15 cm), and Fenpropathrin in strain 3 (1.8 cm). Group 1, which showed the greatest inhibition, comprised Bifenthrin (0.47–0.68 cm), Bifenthrin + Carbosulfan (0–0.88 cm), and Fenpropathrin in strains 1 and 2 (1.37 and 1.38 cm). Pyridaben was also assigned to this group for strains 1 and 2 (1.32 and 1.62 cm). The corresponding mycelial growth inhibition indices (MGI) are shown in Figure 1.

### 3.5. Analysis of Mycelial Growth in Bioassay with Chemical Herbicides

The bioassay with herbicides demonstrated a consistent pattern across the four *T. afroharzianum* strains, showing strong inhibition of mycelial growth by all five evaluated molecules. Cluster analysis revealed clear separation between the groups, with mean silhouette values exceeding 0.74 (Table 5). Only two groups were identified: Cluster 1, consisting of the control (without product application), and Cluster 2, which included all treatments with herbicides. The active ingredients Glyphosate and Trifluralin completely inhibited fungal growth in all strains. For Glufosinate, no growth was observed in strains 1 and 2, while strains 3 and 4 exhibited growth values of 0.48 and 0.42 cm, respectively. The herbicides 2,4-D and Flumioxazine resulted in growth ranging from 0.82 to 1.13 cm and from 0.23 to 0.92 cm, respectively. The mycelial growth inhibition indexes (MGI), presented in Figure 1, were all greater than 79.4%.

### 3.6. Simulation of Spray Solution and Effect of Inputs on Conidial Germination

The second test, conducted to simulate the spray solution and evaluate the effect of the 16 products that demonstrated high inhibition rates of mycelial growth in *T. afroharzianum* strains (Table 6), showed that most of these products (twelve) did not negatively affect the number of germinated conidia, which remained above 76%. The two groups were well separated, with an average silhouette value exceeding 0.86. In contrast, treatments with products based on *B. amyloliquefaciens* (bionematicide) and Picoxystrobin + Benzovindiflupyr (fungicide) resulted in germination rates lower than 8.5% and 14%, respectively. Furthermore, fungicides containing Picoxystrobin + Prothioconazole and Trifloxystrobin + Tebuconazole exhibited strain-specific inhibition, with germination varying between 25.2% and 68.8%, and between 21.2% and 69.3%, respectively.

**Figure 2:**
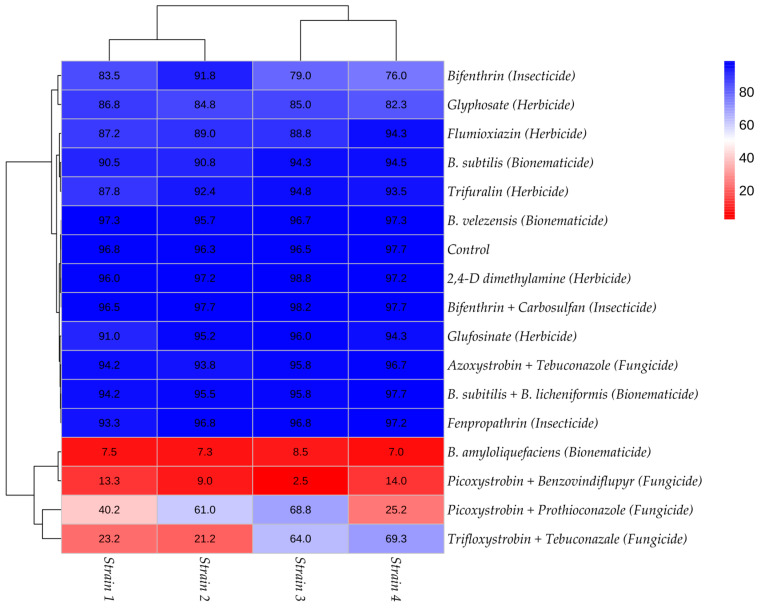
Heatmap constructed based on the conidial germination index observed in the in vitro bioassay simulating the spray solution of sixteen inputs.

The heatmap, constructed based on the average germination rates, illustrates the pattern observed across the four *T. afroharzianum* strains in the second assay with the sixteen products. It reveals that only treatments with *B. amyloliquefaciens* (bionematicide), Picoxystrobin + Benzovindiflupyr (fungicide), Picoxystrobin + Prothioconazole, and Trifloxystrobin + Tebuconazole had a significant impact on conidial germination (Figure 2).

## 4. Discussion

As previously noted, sustainable food systems aim to ensure food security while preserving essential resources for future generations [1]. In this context, plant diseases pose a significant challenge to modern agriculture due to their potential to cause considerable losses in global agricultural productivity [2]. As an alternative to synthetic fungicides, biological control has gained prominence for its effectiveness and lower environmental impact [5], particularly with regard to the *Trichoderma* genus, which is widely employed in this approach [6]. However, despite the widespread use of tank mixtures combining chemical and biological agents, such practices can jeopardize the viability and performance of beneficial microorganisms, thereby diminishing their effectiveness in managing phytopathogens [13,14].

Variable effects were observed among the insecticides tested. Bifenthrin, Bifenthrin + Carbosulfan, and Fenpropathrin were the formulations that most significantly affected the mycelial growth of *T. afroharzianum*, although they did not influence conidial germination. These results partially agree with previous reports for other *Trichoderma* species, yet they also reveal marked interspecific variation. For instance, Mareeswaran and Asir [16] reported that Deltamethrin inhibited 53%, 45%, and 32% of mycelial growth in *T. harzianum*, *T. viride*, and *T. atroviride*, respectively, whereas Bharadwaz et al. [18] observed inhibition levels below 3% for *T. viride* exposed to Deltamethrin and Fenpropathrin. In contrast, our study recorded inhibition rates exceeding 67.3% for *T. afroharzianum*, demonstrating that even insecticides considered compatible with other *Trichoderma* species can exert stronger inhibitory effects depending on the fungal strain. Nonetheless, conidial germination rates above 93.3% under Fenpropathrin treatment suggest that conidia were more tolerant than vegetative mycelia under the tested conditions. Similarly, Acephate exhibited no inhibitory effect, which aligns with the results reported by Ramanagouda and Naik [20] for the same compound.

All herbicidal molecules tested inhibited the mycelial growth of *T. afroharzianum* strains by more than 79.4%, although their effects on conidial germination were considerably lower. These results contrast with those obtained for other *Trichoderma* species. Santoro et al. [27] reported that 2,4-D and Trifluralin reduced the growth of *T. atroviride* by only 28.77% and 31.70%, respectively. In the same study, conidial germination decreased by 10.66%, 12.44%, and 0% for 2,4-D, Glyphosate, and Trifluralin, while in the present study, reductions were 1.2%, 13.2%, and 5.2% for these same molecules, respectively. According to Mareeswaran and Asir [16], Glyphosate inhibited 44%, 33%, and 73% of the mycelial growth of *T. harzianum*, *T. viride*, and *T. atroviride*, respectively. Similarly, Celar and Kos [19] observed inhibition above 75% in *T. asperellum* and *T. gamsii* exposed to dinitroaniline herbicides such as Pendimethalin and Trifluralin. In our study, Glyphosate completely suppressed mycelial growth in all four *T. afroharzianum* strains but did not significantly affect conidial germination, confirming that susceptibility to herbicides is both strain- and species-dependent.

Regarding the biological products evaluated, the compatibility patterns also varied considerably. Studies on the in vitro compatibility between microbiological products or other biological control agents remain limited, and no specific records involving *T. afroharzianum* have been found to date. Available research primarily focuses on co-cultivations, the microbial metabolites produced, and their effects on pathogens and diseases [28,29]. In our assays, several *Bacillus*-based products compromised mycelial growth, although only *B. amyloliquefaciens* significantly reduced conidial germination (below 7%). This observation agrees with Luan et al. [30] and Yi et al. [31], who reported antifungal activity of *B. amyloliquefaciens* lipopeptides against *Bipolaris sorokiniana* and *Fusarium graminearum*. In contrast, products based on *Bradyrhizobium elkanii* and *B. japonicum* did not exhibit antagonism toward *T. afroharzianum*, diverging from the findings of Gobbi and Mattos [32], who observed inhibition of *T. harzianum* and *T. asperellum* by the same nitrogen-fixing bacteria. Similar variability among microbial interactions has been noted by Mahpatra et al. [33] and Braga et al. [34]. Despite methodological differences, these results collectively indicate that compatibility among microbial inoculants is highly context- and strain-dependent.

The contrasting antifungal effects observed between the mycelial growth and conidial germination assays are likely associated with differences in exposure conditions. In the mycelial assay, *T. afroharzianum* strains were continuously exposed to agricultural inputs-amended media, resulting in constant fungicidal pressure during the 48 h incubation period. In contrast, during the conidial assay, spores were in dynamic suspension under agitation, simulating field spray conditions without prolonged contact with the active molecules. Such methodological differences may explain why certain products inhibited vegetative growth but not germination, reinforcing the complementary value of both assays in assessing compatibility.

Among the tested chemical fungicides, four formulations significantly reduced conidial germination: Azoxystrobin + Tebuconazole, Picoxystrobin + Prothioconazole, Trifloxystrobin + Tebuconazole, and Picoxystrobin + Benzovindiflupyr. These combinations involve active ingredients from distinct chemical groups, whose mechanisms of action explain their inhibitory potential. Strobilurins (Group 11, QoI) inhibit mitochondrial respiration at complex III (*cyt-b* gene), triazoles (Group 3, DMI) disrupt ergosterol biosynthesis by inhibiting the *erg11* and *cyp51* genes, and carboxamides (Group 7, SDHI) interfere with cellular respiration by targeting the *sdh* enzyme [35]. The simultaneous disruption of mitochondrial and membrane functions likely accounts for the observed reduction in conidial viability.

Finally, the results demonstrated clear variation in the susceptibility of *T. afroharzianum* strains to some input stressors. Marked differences were observed in mycelial growth in response to the *B. aryabhattai*-based product, mineral oil (adhesive spreader), and the acaricide Pyridaben, as well as in conidial germination under exposure to the fungicidal mixtures Picoxystrobin + Prothioconazole and Trifloxystrobin + Tebuconazole. These strain-dependent differences suggest that susceptibility to agricultural inputs is not only species-specific, as previously reported for other *Trichoderma* species [20,32], but may also vary at the intraspecific level within *T. afroharzianum*. This finding underscores the novelty of this study, as most previous research has focused on interspecific comparisons within the genus *Trichoderma*, whereas this work provides evidence of intraspecific variability. Therefore, these findings reinforce the need to consider strain-specific compatibility when selecting microbial inoculants for agricultural systems that combine biological and chemical inputs.

## 5. Conclusions

This study evaluated the in vitro compatibility of *Trichoderma afroharzianum* strains with a wide range of agricultural inputs, including microbial inoculants, insecticides, herbicides, fungicides, and adjuvants, several of which had not been previously tested in this context. The findings revealed marked variability in susceptibility among strains, indicating that compatibility is influenced not only by the chemical nature of the active compounds but also by strain-specific physiological traits within *T. afroharzianum*.

While certain inputs, particularly some insecticides, herbicides, and microbial products, strongly inhibited mycelial growth, only a few affected conidial germination, especially under conditions simulating field application. Fungicidal mixtures containing strobilurins and triazoles were the most inhibitory, reducing both mycelial growth and conidial viability, which may compromise the field performance of *T. afroharzianum* formulations used in biological control.

These results provide novel insights into the intraspecific variability of chemical tolerance in *T. afroharzianum*, a factor largely overlooked in previous studies focusing on interspecific comparisons within *Trichoderma*. This evidence underscores the importance of strain-level evaluation when selecting biological control agents for integrated pest management systems, particularly where co-application with chemical inputs is anticipated. Future research should aim to validate these findings under field conditions across diverse crops and environmental settings, explore dose-dependent and temporal responses, and assess the combined efficacy of compatible chemical and biological inputs against target phytopathogens, thus contributing to safer and more sustainable crop protection strategies.

## Figures and Tables

**Table 1:** Description of agricultural inputs used in this in vitro compatibility study with Brazilian strains of *Trichoderma afroharzianum.*

Order	Active Ingredient	Input Class	Concentration Used
1	*Bacillus subtilis*	Bionematicide	10^7^ CFU/mL *
2	*Bacillus amyloliquefaciens*	Bionematicide	10^7^ CFU/mL
3	*Bacillus subtilis* + *B. licheniformis*	Bionematicide	10^7^ CFU/mL
4	*Bacillus velezensis*	Bionematicide	10^7^ CFU/mL
5	*Bacillus methylotrophicus*	Bionematicide	10^7^ CFU/mL
6	*Bacillus pumilus*	Bionematicide	10^7^ CFU/mL
7	*Bradyrhizobium elkanii*	Bioinoculant	10^7^ CFU/mL
8	*Bradyrhizobium japonicum*	Bioinoculant	10^7^ CFU/mL
9	*Priestia aryabhattai*	Bioinoculant	10^7^ CFU/mL
10	*Trichoderma harzianum*	Biofungicide	10^7^ conidia/mL
11	Azoxystrobin + Tebuconazole	Fungicide	0.04%
12	*Melaleuca alternifolia* extract (Tea tree)	Fungicide	0.25%
13	Pencycuron	Fungicide	0.40%
14	Picoxystrobin + Benzovindiflupyr	Fungicide	0.60%
15	Picoxystrobin + Prothioconazole	Fungicide	0.50%
16	Trifloxystrobin + Tebuconazole	Fungicide	0.67%
17	Abamectin + Cyantraniliprole	Insecticide/Acaricide	0.19%
18	Acephate	Insecticide	0.25%
19	Azadirachtin	Insecticide/Fungicide	1.00%
20	Bifenthrin	Insecticide	1.33%
21	Bifenthrin + Carbosulfan	Insecticide	6.67%
22	Mineral oil	Adhesive Spreader/Insecticide	0.25%
23	Diafenthiuron	Insecticide	0.40%
24	Fenpropathrin	Insecticide	0.11%
25	Isocycloseram	Insecticide	0.03%
26	Pyridaben	Insecticide/Acaricide	0.38%
27	Tolfenpyrad	Insecticide	0.60%
28	2.4-D dichlorophenoxy + dimethylamine	Herbicide	0.50%
29	Flumioxazin	Herbicide	0.17%
30	Glyphosate	Herbicide	4.00%
31	Glufosinate-Ammonium Salt	Herbicide	0.56%
32	Trifluralin	Herbicide	0.63%

* CFU: Colony Forming Units.

**Table 2:** Statistical analysis, using the silhouette validation method, of mycelial growth (cm) observed in a bioassay with four strains of *Trichoderma afroharzianum* regarding compatibility with ten microbiological products.

Input	Group	MG ^a^	SV ^b^	Group	MG	SV
	Strain 1 (0.84) ^c^			Strain 2 (0.77)		
*B. amyloliquefaciens*	1	0.00 ± 0.00	0.69	1	0.00 ± 0.00	0.78
*P. aryabhattai*	2	3.77 ± 2.08	0.77	2	3.85 ± 1.81	0.15
*B. elkanii*	3	5.50 ± 0.00	1.00	3	5.50 ± 0.00	1.00
*B. japonicum*	3	5.50 ± 0.00	1.00	3	5.50 ± 0.00	1.00
*B. methylotrophicus*	2	3.32 ± 0.75	0.77	2	2.33 ± 1.18	0.15
*B. pumilus*	3	5.50 ± 0.00	1.00	3	5.50 ± 0.00	1.00
*B. subtilis*	1	0.17 ± 0.26	0.69	1	0.13 ± 0.33	0.78
*B. subtilis + B. licheniformis*	1	0.65 ± 0.67	0.69	1	0.30 ± 0.46	0.78
*B. velezensis*	1	1.48 ± 1.63	0.69	1	1.05 ± 1.38	0.78
*T. harzianum*	3	5.50 ± 0.00	1.00	3	5.50 ± 0.00	1.00
Control	3	5.50 ± 0.00	1.00	3	5.50 ± 0.00	1.00
	Strain 3 (0.77)			Strain 4 (0.78)		
*B. amyloliquefaciens*	1	0.00 ± 0.00	0.82	1	0.00 ± 0.00	0.94
*P. aryabhattai*	3	5.12 ± 0.44	0.86	3	5.02 ± 0.54	0.81
*B. elkanii*	3	5.50 ± 0.00	0.86	3	5.50 ± 0.00	0.81
*B. japonicum*	3	5.18 ± 0.35	0.86	3	5.50 ± 0.00	0.81
*B. methylotrophicus*	2	3.83 ± 0.38	0.00	2	4.17 ± 0.40	0.00
*B. pumilus*	3	5.33 ± 0.19	0.86	3	5.28 ± 0.25	0.81
*B. subtilis*	1	0.00 ± 0.00	0.82	1	0.00 ± 0.00	0.94
*B. subtilis + B. licheniformis*	1	0.67 ± 1.03	0.82	1	0.45 ± 0.60	0.94
*B. velezensis*	1	0.98 ± 1.08	0.82	1	0.00 ± 0.00	0.94
*T. harzianum*	3	5.50 ± 0.00	0.86	3	5.48 ± 0.04	0.81
Control	3	5.50 ± 0.00	0.86	3	5.50 ± 0.00	0.81

^a^ Micelial Growth ± Standard Deviation, ^b^ Silhouette Validation, ^c^ Average silhouette.

**Table 3:** Statistical analysis, using the silhouette validation method, of mycelial growth (cm) observed in a bioassay with four strains of *Trichoderma afroharzianum* regarding compatibility with six chemical fungicides.

Input	Group	MG ^a^	SV ^b^	Group	MG	SV
	Strain 1 (0.84) ^c^			Strain 2 (0.84)		
Azoxystrobin + Tebuconazole	1	0.23 ± 0.26	0.97	1	0.25 ± 0.27	0.96
*Melaleuca alternifolia* extract	2	4.75 ± 0.84	0.00	2	4.72 ± 0.86	0.00
Pencycuron	3	5.50 ± 0.00	1.00	3	5.50 ± 0.00	1.00
Picoxystrobin + Benzovindiflupyr	1	0.00 ± 0.00	0.97	1	0.00 ± 0.00	0.96
Picoxystrobin + Prothioconazole	1	0.00 ± 0.00	0.97	1	0.27 ± 0.29	0.96
Trifloxystrobin + Tebuconazole	1	0.00 ± 0.00	0.97	1	0.00 ± 0.00	0.96
Control	3	5.50 ± 0.00	1.00	3	5.50 ± 0.00	1.00
	Strain 4 (0.84)			Strain 3 (0.84)		
Azoxystrobin + Tebuconazole	1	0.25 ± 0.30	0.96	1	0.13 ± 0.33	0.97
*Melaleuca alternifolia* extract	2	4.63 ± 0.95	0.00	2	4.72 ± 0.86	0.00
Pencycuron	3	5.50 ± 0.00	1.00	3	5.50 ± 0.00	1.00
Picoxystrobin + Benzovindiflupyr	1	0.23 ± 0.27	0.96	1	0.27 ± 0.41	0.97
Picoxystrobin + Prothioconazole	1	0.33 ± 0.37	0.96	1	0.08 ± 0.20	0.97
Trifloxystrobin + Tebuconazole	1	0.00 ± 0.00	0.96	1	0.00 ± 0.00	0.97
Control	3	5.50 ± 0.00	1.00	3	5.50 ± 0.00	1.00

^a^ Micelial Growth ± Standard Deviation, ^b^ Silhouette Validation, ^c^ Average silhouette.

**Table 4:** Statistical analysis, using the silhouette validation method, of mycelial growth (cm) observed in a bioassay with four strains of *Trichoderma afroharzianum* regarding compatibility with eleven insecticides.

Insecticides	Group	MG ^a^	SV ^b^	Group	MG	SV
	Strain 1 (0.85) ^c^			Strain 2 (0.86)		
Abamectin + Cyantraniliprole	3	5.12 ± 0.42	0.84	3	5.50 ± 0.00	0.97
Acephate	3	5.50 ± 0.00	0.84	3	5.50 ± 0.00	0.97
Azadirachtin	3	5.50 ± 0.00	0.84	3	5.50 ± 0.00	0.97
Bifenthrin	1	0.68 ± 0.12	0.88	1	0.68 ± 0.40	0.83
Bifenthrin + Carbosulfan	1	0.88 ± 0.26	0.88	1	0.88 ± 0.20	0.83
Adhesive spreader/Insecticide	2	4.55 ± 1.05	0.84	2	4.68 ± 0.90	0.57
Diafenthiuron	3	5.50 ± 0.00	0.84	3	5.50 ± 0.00	0.97
Fenpropathrin	1	1.37 ± 0.50	0.88	1	1.38 ± 0.48	0.83
Isocycloseram	3	5.50 ± 0.00	0.84	3	5.42 ± 0.13	0.97
Pyridaben	1	1.32 ± 0.71	0.88	1	1.62 ± 0.83	0.83
Tolfenpyrad	2	4.40 ± 1.21	0.84	2	4.27 ± 1.35	0.57
Control	3	5.50 ± 0.00	0.84	3	5.50 ± 0.00	0.97
	Strain 3 (0.65)			Strain 4 (0.84)		
Abamectin + Cyantraniliprole	3	5.10 ± 0.44	0.86	3	4.92 ± 0.67	0.69
Acephate	3	5.50 ± 0.00	0.86	3	5.50 ± 0.00	0.69
Azadirachtin	3	5.23 ± 0.29	0.86	3	5.10 ± 0.44	0.69
Bifenthrin	1	0.58 ± 0.33	0.56	1	0.47 ± 0.61	0.73
Bifenthrin + Carbosulfan	1	0.00 ± 0.00	0.56	1	0.67 ± 0.75	0.73
Adhesive spreader/Insecticide	2	4.03 ± 0.85	0.40	2	4.05 ± 0.86	0.37
Diafenthiuron	3	5.50 ± 0.00	0.86	3	5.50 ± 0.00	0.69
Fenpropathrin	2	1.80 ± 0.45	0.56	1	1.48 ± 0.74	0.73
Isocycloseram	3	5.23 ± 0.29	0.86	3	5.12 ± 0.58	0.69
Pyridaben	2	3.17 ± 1.41	0.40	2	3.15 ± 1.26	0.37
Tolfenpyrad	2	4.40 ± 1.21	0.40	3	4.52 ± 1.09	0.69
Control	3	5.50 ± 0.00	0.86	3	5.50 ± 0.00	0.69

^a^ Micelial Growth ± Standard Deviation, ^b^ Silhouette Validation, ^c^ Average silhouette.

**Table 5:** Statistical analysis, using the silhouette validation method, of mycelial growth (cm) observed in a bioassay with four strains of *Trichoderma afroharzianum* regarding compatibility with five herbicides.

Herbicide	Group	MG ^a^	SV ^b^	Group	MG	SV
	Strain 1 (0.77) ^c^			Strain 2 (0.75)		
2,4-D	1	0.82 ± 0.21	0.93	1	0.82 ± 0.15	0.89
Flumioxazin	1	0.25 ± 0.28	0.93	1	0.92 ± 1.19	0.89
Glyphosate	1	0.00 ± 0.00	0.93	1	0.00 ± 0.00	0.89
Glufosinate	1	0.00 ± 0.00	0.93	1	0.00 ± 0.00	0.89
Trifluralin	1	0.00 ± 0.00	0.93	1	0.00 ± 0.00	0.89
Control	2	5.50 ± 0.00	0.00	2	5.50 ± 0.00	0.00
	Strain 4 (0.75)			Strain 3 (0.74)		
2,4-D	1	1.00 ± 0.34	0.90	1	1.13 ± 0.28	0.89
Flumioxazin	1	0.23 ± 0.26	0.90	1	0.27 ± 0.31	0.89
Glyphosate	1	0.00 ± 0.00	0.90	1	0.00 ± 0.00	0.89
Glufosinate	1	0.48 ± 0.53	0.90	1	0.42 ± 0.48	0.89
Trifluralin	1	0.00 ± 0.00	0.90	1	0.00 ± 0.00	0.89
Control	2	5.50 ± 0.00	0.00	2	5.50 ± 0.00	0.00

^a ^Micelial Growth ± Standard Deviation, ^b^ Silhouette Validation, ^c^ Average silhouette.

**Table 6:** Statistical analysis, using the silhouette validation method, performed on conidial germination observed in a bioassay simulating the spray solution with four strains of *Trichoderma afroharzianum* and sixteen chemical and biological pesticides.

Input	Group	MG ^a^	SV ^b^	Group	MG	SV
	Strain 1 (0.88) ^c^			Strain 2 (0.88)		
P1 ^d^	1	90.50 ± 09.14	0.94	1	90.83 ± 05.12	0.92
P2	2	07.50 ± 03.08	0.79	2	07.33 ± 02.34	0.91
P3	1	94.17 ± 03.87	0.94	1	95.50 ± 01.87	0.93
P4	1	97.33 ± 01.21	0.92	1	95.67 ± 01.51	0.93
P5	1	94.17 ± 04.22	0.94	1	93.83 ± 01.94	0.93
P6	2	13.33 ± 12.45	0.82	2	09.00 ± 04.47	0.92
P7	2	40.17 ± 26.17	0.51	1	61.00 ± 26.47	0.33
P8	2	23.17 ± 17.08	0.79	2	21.17 ± 12.62	0.81
P9	1	83.50 ± 05.24	0.85	1	91.83 ± 05.53	0.93
P10	1	96.50 ± 02.17	0.93	1	97.67 ± 01.03	0.92
P11	1	93.33 ± 01.53	0.94	1	96.83 ± 00.75	0.93
P12	1	96.00 ± 01.10	0.94	1	97.17 ± 01.17	0.92
P13	1	87.17 ± 03.92	0.91	1	89.00 ± 05.22	0.90
P14	1	86.83 ±0 2.99	0.91	1	84.83 ± 06.71	0.85
P15	1	91.00 ± 04.38	0.94	1	95.17 ± 00.75	0.93
P16	1	87.83 ± 02.40	0.92	1	92.40 ± 02.97	0.93
Test	1	96.75 ± 04.73	0.93	1	96.33 ± 04.38	0.93
Input	Group	MG ^a^	SV ^b^	Grupo	MG	SV
	Strain 3 (0.86) ^c^			Strain 4 (0.87)		
P1 ^d^	1	94.33 ± 02.25	0.91	1	94.50 ± 02.74	0.93
P2	2	08.50 ± 03.94	0.93	2	07.00 ± 03.29	0.85
P3	1	95.83 ± 02.79	0.92	1	97.67 ± 01.03	0.92
P4	1	96.67 ± 01.37	0.92	1	97.33 ± 00.82	0.93
P5	1	95.83 ± 04.31	0.92	1	96.67 ± 01.75	0.93
P6	2	02.50 ± 02.17	0.93	2	14.00 ± 10.16	0.88
P7	1	68.83 ± 23.44	0.63	2	25.17 ± 13.45	0.78
P8	1	64.00 ± 23.64	0.52	1	69.33 ± 25.58	0.55
P9	1	79.00 ± 19.82	0.79	1	76.00 ± 19.86	0.70
P10	1	98.17 ± 01.17	0.90	1	97.67 ± 01.97	0.92
P11	1	96.83 ± 01.47	0.91	1	97.17 ± 02.56	0.93
P12	1	98.83 ± 00.75	0.90	1	97.17 ± 02.04	0.93
P13	1	88.83 ± 05.53	0.89	1	94.33 ± 04.41	0.92
P14	1	85.00 ± 09.67	0.86	1	82.33 ± 11.41	0.80
P15	1	96.00 ± 01.26	0.92	1	94.33 ± 01.75	0.92
P16	1	94.83 ± 01.94	0.92	1	93.50 ± 01.64	0.92
Test	1	96.50 ± 04.96	0.92	1	97.67 ± 02.23	0.92

^a^ Mycelial growth ± Standard Deviation, ^b^ Silhouette Validation, ^c^ Average Silhouette, ^d^ Bionematicides: P1: *B. subtilis*, P2: *B. amyloliquefaciens*, P3: *B. subtilis* + *B. licheniformis*, P4: *B. velezensis*; Fungicides: P5: Azoxystrobin + Tebuconazole, P6: Picoxystrobin + Benzovindiflupyr, P7: Picoxystrobin + Prothioconazole, P8: Trifloxystrobin + Tebuconazole; Insecticides: P9: Bifenthrin, P10: Bifenthrin + Carbosulfan, P11: Fenpropathrin; Herbicides: P12: 2,4-D dimethylamine, P13: Flumioxazine, P14: Glyphosate, P15: Glufosinate and P16: Trifluralin.

## Data Availability

The original contributions presented in the study are included in the article, further inquiries can be directed to the corresponding author.
